# Skin swabbing protocol to collect DNA samples from small-bodied fish species

**DOI:** 10.12688/f1000research.73115.1

**Published:** 2021-10-19

**Authors:** Ceinwen Tilley, Iain Barber, William Norton

**Affiliations:** 1 Neuroscience, Psychology and Behaviour, University of Leicester, Leicester, Leicestershire, LE1 7RH, UK; 2 School of Animal and Rural Sciences, Nottingham Trent University, Nottingham, NG25 0QF, UK; 3 Genetics and Genome Biology, University of Leicester, Leicester, Leicestershire, LE1 7RH, UK; 4 Institute of Biology, Eotvos Lorand University, Budapest, Hungary

**Keywords:** Zebrafish, stickleback, skin swabbing, fin clipping, DNA extraction, refinement, reduction

## Abstract

Fish species are commonly used as experimental models in the laboratory. DNA is routinely collected from these animals to permit identification of their genotype. The current standard procedure to sample DNA is fin clipping, which involves anaesthetising individuals and removing a portion of the caudal fin. While fin clipping reliably generates good quality DNA samples for downstream applications, there is evidence that it can alter health and welfare, leading to infection and impacting on the fish’s behaviour. This in turn can result in greater variation in the data collected. In a recent study we adapted a skin swabbing protocol to collect DNA from small-bodied fish, including sticklebacks and zebrafish, without the use of anaesthetics or sharp instruments. A rayon-tipped swab was used to collect mucus from the flank of the fish, which was then used for DNA extraction. We subsequently demonstrated that compared to fin clipping, skin swabbing triggered fewer changes in stress axis activation and behaviour. We also found that data collected from fish that had been swabbed were less variable than data from fish that had been fin clipped, potentially allowing smaller sample sizes in experimental groups after using this technique, and thereby reducing animal use. Here we provide a detailed protocol explaining how to collect DNA samples from small laboratory fish using skin swabs.

Research highlights **Scientific benefits**

Skin swabbing causes less variation in subsequent data collection compared to fin clipping. This can improve the quality of data collection with the potential to aid comparison of results across laboratories.

**3Rs benefits**

DNA collection by skin swabbing causes fewer changes to stress axis activation and behaviour than fin clipping, the current standard technique. Skin swabbing also removes the need to use anaesthetic, and decreases the potential for fish to experience pain or infection compared to removal of tissue by fin clipping. In addition, the smaller variation in data collection caused by skin swabbing compared to fin clipping means that fewer animals need to be included in experiments.

**Practical benefits**

Skin swabbing is quick to perform, relatively non-invasive for the fish and collects DNA of suitable quality for PCR amplification. In addition, skin swabbing does not require sharp scalpels, reducing the possibility of harm to researchers.

**Current applications**

Skin swabbing can be used to collect DNA samples from fish species with a body size that is greater than 20 mm, the smallest body size that we could swab without risk harm to the animal. It is particularly useful when genotype information is needed from fish, such as identifying transgenic or mutant carriers in a group of animals.

**Potential applications**

Skin swabbing should be suitable to collect DNA from any species with a mucus layer on the body, including fish, frogs and toads. The technique may also be suitable for automation in the future.

## Introduction

Model fish species including zebrafish and sticklebacks are used frequently for experiments in the laboratory. Some of the advantages of these species include their small size, short life cycle and ease of maintenance, making it easy to keep many fish within laboratory aquaria. Fish also tend to be easy to manipulate genetically, and display similarities to other vertebrates permitting data to be translated across species ( Bert *et al*., 2016; Schaeck *et al*., 2013; NC3Rs, 2014; Flecknell, 2002). Fish are used to study a range of disciplines including developmental biology, ecology, neuroscience and behaviour. They are also used as models for aspects of human disease including cancer ( Hason and Bartunek, 2019), visual impairments ( Richardson *et al*., 2017) and neurological disorders ( Kalueff *et al*., 2014; Norton, 2013). The recent development of techniques to manipulate the genome, including transgenesis ( Higashijima, 2008), optogenetics ( Wyart and Del Bene, 2011) and CRISPR-Cas9 mutagenesis ( Cornet *et al*., 2018; Albadri *et al*., 2017) means that DNA needs to be collected from many of these animals to facilitate identification of their genotype. In addition, the number of fish used in experiments is increasing each year. For example, in 2015, 14% of all regulated animal procedures in Britain were undertaken on fish (Home Office, 2014) and this rose to 30% in 2019, which represents more than one million procedures in the UK alone ( Home Office, 2019). Accordingly, the number of fish undergoing DNA sampling is also likely to rise over time.

The current standard procedure to sample DNA is fin clipping under non-terminal anaesthesia ( Xing *et al*., 2014). Typically, animals are immersed in anaesthetic until they become unresponsive to touch. A small part of the caudal fin is removed using a sterile scalpel before fish are allowed to recover in fresh system water. In some cases, pre-operative analgesia is applied by immersing the fish in water containing lidocaine ( Schroeder and Sneddon, 2017). A recent survey indicated that 85% of zebrafish labs use fin clipping to collect DNA ( Lidster *et al*., 2017). However, despite its popularity, there is evidence that fin clipping can alter health and welfare, leading to infection and altering growth and survival ( Taslima *et al*., 2015). Fish display signs of pain after fin clipping ( Deakin *et al*., 2019), as well as alterations to anxiety-like behaviour, including increased ventilation ( Schroeder and Sneddon, 2017), reduced activity ( Deakin *et al*., 2019; Schroeder and Sneddon, 2017), increased time at the bottom of a tank ( Deakin *et al*., 2019; De Lombaert *et al*., 2017; Schroeder and Sneddon, 2017; White *et al*., 2017) and decreased feeding (De Lombaert *et al*., 2017). Fin clipping can also trigger the release of the primary stress hormone cortisol ( White *et al*., 2017). This suggests that there is a need for alternative, more refined techniques to collect DNA from model fish species.

Our recent research has shown that skin swabbing – collecting mucus from the flank of a fish and extracting DNA from it ( Breacker *et al*., 2017) – provides a suitable alternative to fin clipping in small laboratory fish. The skin swabbing protocol had already been used to collect DNA from many fish species ( Sebire *et al*., 2015; Taslima *et al*., 2015; Mirimin *et al*., 2011; Le Vin *et al*., 2011; Campanella and Smalley, 2006) including sticklebacks (Sebire *et al*., 2017), and we adapted it for use in zebrafish ( Tilley *et al*., 2020; Breacker *et al*., 2017). In this methods paper, we describe how to swab and extract DNA from fish mucus in a step-by-step manner. We also present data from our recent research ( Breacker *et al*., 2017; Tilley *et al*., 2020) showing that skin swabbing is a refinement compared to fin clipping, with the added potential to reduce the number of animals used in experiments.

## Methods

### Ethical approval

All work was conducted under a UK Home Office licence to Dr Norton (licence no. P8F9CCE8B), and was approved by a local Animal Welfare and Ethical Review Body (AWERB) committee at the University of Leicester.

### Maintaining fish in the laboratory

*Threespined sticklebacks*

Stocks of F2 generation lab-bred three-spined sticklebacks ( *Gasterosteus aculeatus*) were generated by *in vitro* fertilisation in July 2017 ( Barber and Arnott, 2000). The parental background was a wild freshwater population originally collected from the River Welland (Market Harborough, Leicestershire, UK) in 2015. Adult sticklebacks were fed an *ad libitum* diet of defrosted bloodworm ( *Chironomus* sp. larvae). Groups of fish were pooled into large stock tanks (27 L) in a dedicated fish facility at the University of Leicester. The choice of which groups to mix was haphazard, matching fish of similar size and age. We did not expect this to cause changes in behaviour such as aggression, based upon our previous research into adult fish behaviour ( Norton *et al.*, 2011). The tanks housed 40 fish on a re-circulating system (Xenoplus systems, Techniplast) with a flow rate of two tank changes per hour. The system water was made from reverse osmosis water with Instant Ocean marine salts added (Aquarium Systems, UK). The water parameters were pH ~7.1, 0 ppm ammonia, 0 ppm nitrate, ~4 ppm nitrite and ~4000 DKH conductivity. Temperature and light–dark conditions were adjusted periodically to simulate natural seasonal variation until March 2018. Fish were then kept at 12±1°C on a 12:12 h light:dark cycle (i.e. March conditions) to maintain non-breeding conditions for the duration of the experiments, which were conducted from May 2018 onwards. 567 adult sticklebacks were used to develop this protocol, with a mean (± standard deviation) length of 36.74 mm (±2.98 mm) and a mean weight of 0.67 g (±0.2 g). All experimental studies were carried out using non-reproductive individuals, avoiding confounding factors associated with territorial and courtship behaviours. This means that although the sticklebacks were about one year old and adult size and weight, they had not developed sexually because they had not been exposed to spring light conditions and temperatures.

*Zebrafish*

Adult AB wild-type zebrafish ( *Danio rerio*) were generated from stock maintained at the University of Leicester. Fish were fed *ad libitum* each afternoon at the end of the experiments (Zebrafeed (Sparos)). The animals used in this study were arbitrarily netted from a group of 40 fish in 8 L tanks on a re-circulating system (ZebTEC multi-link water treatment unit, Techniplast) with a flow rate of 7.6 L per tank per hour (ca. one tank change per hour). The system water was made from reverse osmosis water with Instant Ocean salts (Aquarium Systems, UK) added. The water parameters were pH ~7.1, 0 ppm ammonia, 0 ppm nitrate, ~4 ppm nitrite and ~525 DKH conductivity. The fish were maintained at 28±1°C and in a 14:10 h light:dark cycle, standard lighting conditions for laboratory-housed zebrafish. The following zebrafish strains were used; AB wild-type and *Tg* ( *Vmat2:GFP*) (Wen *et al*., 2008). The zebrafish used to develop this protocol (n = 630) had a mean (± s.d.) length of 34.99 mm (±1.66 mm) and a mean weight of 0.38g (±0.08 g) and included a mix of sexually mature males and females (approximate ratio 1.5:1 males:females) between three and six months old.

No enrichment was provided, as is standard procedure in our fish facility. No fish of either species died during these experiments, and all animals were killed by a Schedule 1 procedure at the end of this study.

### Fin clipping sticklebacks and zebrafish

This experiment was first reported in Breacker *et al*. (2017). To collect DNA by fin clipping, a single fish was removed from its home tank using a small hand net. The fish was pre-treated with an anaesthetic by placing it in a tank containing 168 mg/L ethyl 3-aminobenzoatemethanesulfonate (MS-222) buffered to pH 7.2 with sodium bicarbonate dissolved in fresh system water. Once the fish was no longer responsive to touch, it was gently caught in a net and placed into a Petri dish containing a small amount of water. Fins were clipped using a sterile razor blade taking care to only remove about one third of the caudal fin. The excised fin tissue was placed into a sterile labelled Eppendorf tube, and the fish was moved to a recovery tank containing system water. The fish’s behaviour was monitored until it had recovered consciousness, so that it swam around in the tank freely. Fish were placed into individual holding tanks until DNA extraction and identification was complete. Fin clip DNA was extracted using the same DNA extraction buffer as for skin swabbing (see section 4.1.2. below), but with the addition of 15 μl of 20 mg/ml Proteinase K. This was incubated at 57°C for 30 minutes, followed by addition of 400 μl chilled isopropanol. The solutions were mixed and the DNA solution was then chilled at −80°C for 30 minutes. The solution was centrifuged for 10 minutes at 10,625 g, the supernatant decanted, and the remaining pellet washed with 190 μl 70% EtOH. After a further centrifugation step (two minutes at 10,625 g) the DNA pellet was air dried and resuspended in 30 μl ddH _2_O.

### Skin swabbing sticklebacks and zebrafish

To collect DNA by skin swabbing, a single fish was removed from its home tank using a small hand net. The fish was pre-treated with an analgesic by placing it in a tank containing 2 mg/L lidocaine dissolved in fresh system water. The fish was immersed in this solution for 45 minutes immediately before skin swabbing was carried out. The fish was then re-caught in the net, and gently restrained on top of a wetted sponge. The uppermost surface of the fish was exposed to the air to permit DNA collection. A sterile swab was gently stroked along the flank of the fish, from head to tail, five to ten times. The swab was placed into a clean labelled Eppendorf tube, and the fish was placed into a holding tank until DNA extraction and identification was complete. The DNA was extracted from the swab by adding DNA extraction buffer warmed to 55°C and letting it incubate for two minutes. The swab was then removed, taking care to squeeze out as much liquid as possible. The DNA was precipitated by addition of 400 μl chilled isopropanol. The solutions were mixed and the DNA solution was chilled at -80°C for 30 minutes. The solution was centrifuged for 10 minutes at maximum speed (10,625 g), the supernatant decanted, and the remaining pellet washed with 190 μl 70% EtOH. After a further centrifugation step (two minutes at 10,625 g) the DNA pellet was air dried and resuspended in 30 μl ddH _2_O.

For experiments comparing skin swabbing to fin clipping in the same animal, fish were first swabbed and then fin clipped immediately afterwards. No lidocaine was used in these experiments, which were performed before we investigated whether pain relief could improve the skin swabbing protocol. Their recovery and welfare was monitored by looking for changes in balance, locomotion or respiration in the hour after the procedure was completed.

### Behavioural analysis

Adult sticklebacks and zebrafish were tested for changes in locomotion and anxiety-like behaviour in the open field test, novel tank diving test and black and white test as described in Norton and Carreño Gutiérrez (2019). Fish were filmed for five minutes the side (novel tank diving test) or above (open filed and black and white tests). In the novel tank test, we compared distance swum, and the time spent in the top or bottom half of a novel trapezoid-shaped tank. An anxious fish should prefer to remain close to the bottom of the tank. In the open field test we measured distance swum, and time spent at the side or in the centre of a novel tank. An anxious fish should prefer to swim close to the side of the tank. In the black and white test, we analysed the preference to spend time on the black or white side of the tank. A more anxious fish should prefer to stay on the black side of the tank. Behaviour was then analysed using videotracking software from ViewPoint Lifesciences.

### PCR amplification of genes from stickleback and zebrafish

The DNA samples obtained from three-spined sticklebacks were amplify the sex-linked molecular marker Isocitrate dehydrogenase (IDH) using the following primers: STKSEX forward primer 5′ GGGACGAGCAAGATTTATTGG 3′; STKSEX reverse primer 5′ TATAGTTAGCCAGGAGATGG 3′. Females produce a single band of approximately 300 bp, whilst males produce two products of 270 bp and 300 bp. 10 μl PCR reactions were set up using 5 μl Red Taq master mix (Sigma-Aldrich, UK), 0.5 μl of the forward and reverse primer, 3 μl of DNA template and 1 μl ddH _2_O). The PCR conditions were 94°C for 5 min, followed by 40 cycles of: 95°C for 30 sec, 56°C for 30 sec, 72°C for 30 sec, with a final extension of 72°C for 10 min. PCR products were visualised on a 5% agarose gel. PCR reactions were run on a Veriti 96 well thermal cycler.

For zebrafish, PCR was used to identify AB WT and transgenic *Tg* ( *Vmat2:GFP*) fish. This used primers designed against the genes *microphthalmia-associated transcription factor a* (for WT) and *green fluorescent protein* for *Tg (Vmat2:eGFP).* The primers used were: *mitfa* forward primer 5′ GCCAACTAAATTTCATGAACC 3′; reverse primer 5′ AAATCAACTAATTGTTTACACG 3′as described by Lister *et al.* (1999) and GFP forward 5′ TCGAGCTGGACGGCGACGT 3′; reverse 5′ GGTGCTCAGGTAGTGGTTGTC 3′. 10 μl reactions were set up (5 μl Red Taq master mix (Sigma-Aldrich, UK), 0.5 μl of the forward and reverse primer 3 μl of DNA template and 1μl ddH _2_O). The PCR conditions were 94°C for 2 min, followed by 35 cycles of: 94°C for 30 sec, 60°C for 30 sec, 72°C for 1 min, with a final extension of 72°C for 10 min. Products were visualised on a 2% agarose gel. PCR reactions were run on a Veriti 96 well thermal cycler.

### Cortisol extraction and quantification.

Cortisol was extracted from the water samples by pumping it through Sep-pak Plus C18 solid phase extraction cartridges (Waters Ltd., UK) following the protocol developed by the Cefas Weymouth Laboratory ( Ellis *et al.,* 2004). Cartridges were primed with 5 ml of methanol followed by 5 ml of distilled water (dH20) and water samples were pumped through the cartridges at 5 ml/min. Each cartridge was washed with 5 ml of dH20, then air-dried, wrapped in Parafilm ^®^ and stored at −80 °C until elution with 5 ml ethyl acetate. For the quantification by radioimmunoassay the eluted extracts were evaporated at 45°C under nitrogen and each residue was reconstituted in 500 μl of RIA buffer until assayed. The elution and the quantification were carried out in a blind manner to reduce bias when analysing the data.

### Statistical analysis

Statistical analyses were carried out using GraphPad Prism7. Data were tested for normality using the using the Shapiro–Wilk test. Since the majority of data were not distributed normally, we analysed all data using the non-parametric Kruskal–Wallis test followed by a Dunn's multiple comparisons test comparing each treatment to the control group.

## Detailed skin swabbing protocol

### Equipment needed

Please refer to Videos 1 and 2 to see how the DNA collection part of this protocol can be carried out.

• Fish for DNA sampling, for example available from ZIRC or similar organisations.
• Two aquariums large enough to hold a single fish, e.g. Techniplast ZebTEC 1.1 L tank or similar, e.g. Techniplast ZB300BF.
• Access to fresh water from the main aquarium system.
• A small sponge to rest fish on during swabbing, e.g. Vitrex 10 2904 square sponge or similar, available online. We use a sponge measuring approximately 10 cm length × 5 cm width × 3 cm depth. We cut a small 0.5 cm groove into the upper surface of the sponge to make it easier to restrain the fish.
• A clean net large enough to comfortably catch and restrain a single fish, e.g. Marina Fine Soft Mesh Fish Net with Plastic Coated Handle or similar, available online. The net can be rinsed with aquarium water in between use.
• A sterile rayon-tipped swab such as a cotton bud or a similar commercially available product/. Cotton buds should be autoclaved and dried before use to sterilise them.
• A sterile 1.5 ml Eppendorf tube, labelled with a name or number to identify the fish e.g. Sigma Aldrich Ref. EP0030120086. RRID:SCR_000786.
• Clean scissors, suitable to cut the stem of a sterile swab e.g. Brabantia 121746 Tasty+ Kitchen Scissors, or similar, available online.
• Lidocaine hydrochloride e.g. Sigma Aldrich Ref. PHT1257.
• DNA extraction solution (see preparation of reagents below).
• Isopropanol (also known as 2-Propanol) e.g. Sigma Aldrich Ref. I9516.
• Absolute ethanol e.g. Sigma Aldrich Ref. 51976.
• Trizma Base (TRIS) e.g. Sigma Aldrich Ref. 648310-M.
• Ethylenediaminetetraacetic acid (EDTA) e.g. Sigma Aldrich Ref. EDS-100G.
• Sodium chloride (NaCl) e.g. Sigma Aldrich Ref. S7563.
• Sodium dodecyl sulphate (SDS) e.g. Sigma Aldrich Ref. L3771.

### Preparation of reagents

*Prepare stock solutions to make the DNA extraction solution:*

• 1 M TRIS pH 7.5
• 0.5 M EDTA
• 2 M NaCl
• 10% SDS

*Make 100 ml DNA extraction buffer from the stock solutions.*

Mix:

• 20 ml 1 M TRIS pH 7.5
• 5 ml 0.5 M EDTA
• 12.5 ml 2 M NaCl
• 57.5 ml dH _2_O

Autoclave the individual stock solutions before use. Once autoclaved, add 5 ml 10% SDS (do not autoclave once SDS has been added). This solution can be stored at room temperature for several weeks until signs of precipitation or contamination become evident.

Make a 70% ethanol solution by adding 3 ml ultrapure water, distilled water or similar to 7 ml absolute ethanol.

Make a solution of 2 mg/L Lidocaine to use for pre-swabbing analgesia. Make a stock by dissolving 2 g lidocaine in 1 ml ultrapure water. Add 1 ml stock solution to 1 L system water to generate a final concentration of 2 mg/L.

*Set up equipment in the laboratory*

Label one 1.5 ml Eppendorf tube with a name or number to identify the fish to be swabbed.

Make sure that you have one sterile swab for each animal.

Label enough holding tanks to maintain each fish separately until identification is complete. These tanks should be placed onto the main aquarium system so that fish have access to flowing oxygenated water.

Fill a small tank with system water up to depth of 1 cm or 2 cm (the swabbing tank). Place the grooved sponge into this water, groove side up, making sure that it is completely wet. The top of the sponge should not be covered in water, so that the exposed flank of the fish can be swabbed easily without getting the swab too wet.

Place the group of fish that you wish to identify into a holding tank close to the swabbing tank.

Set up a tank containing 2 mg/L lidocaine for pre-operative analgesia/pain relief.

*Apply pain relief before DNA collection*

Administer lidocaine prior to swabbing to provide pain relief. Immerse the group of fish in a solution of 2 mg/L lidocaine for 45 minutes prior to swabbing. The fish require 45 minutes for the lidocaine to take effect, and swabbing should be carried out immediately afterwards.

*Swab the fish to collect DNA*

Pre-warm an aliquot of DNA extraction solution to 55°C; chill the isopropanol to −20°C.

Gently catch a single fish from the analgesia tank in a net and transfer it to the swabbing tank containing the sponge. Use the net to restrain the fish on top of the wetted sponge, positioning the body into the groove. Using thumb and forefinger hold the net so that the side of the fish against the sponge rests on the net, whereas the uppermost side of the fish is exposed for swabbing. The underside of the fish rests on the net, not the sponge, thereby minimising contamination for the sponge between different fish (Video 1).

Using a sterile rayon-tipped swab (such as a cotton bud or similar), gently stroke the fish five to ten times from the operculum to the base of the caudal fin. Very little pressure is required to collect enough mucus for DNA extraction. Be as gentle as possible to avoid damaging the animal. The whole procedure – netting, swabbing and returning the animal to the tank should only take around 30 seconds (Video 2).

Swabbing videos 1 and 2 Preparing the zebrafish for swabbing and carrying out the DNA extraction. Copyright: © 2021 Tilley C et al. 2021

Place the swab into a labelled 1.5 ml Eppendorf tube. The handle of the swab can be cut off using scissors, and the tip stored at room temperature (either dry or in DNA extraction solution) with the lid of the tube closed. ^1^

Place each fish into a labelled holding tank on the aquarium system. Monitor its health and welfare, including locomotion, balance and respiration, in the immediate recovery period afterwards. The fish will remain in this tank until identification is complete. ^2^ Typically it takes half a day to extract DNA, run a PCR and identify the fish. Therefore, fish may be held in single tanks for a few hours up to overnight.

*DNA extraction from swabs* ^3^

• Check that the DNA extraction buffer has been pre-warmed to 55°C.
• Add 400 μl DNA extraction buffer into a 1.5 ml Eppendorf tube containing the swab.
• Incubate the swab at room temperature for at least two minutes.
• Remove the swab using fingers or forceps, and squeeze it against the side of the tube to retain as much extraction solution as possible.
• Add 400 μl of pre-chilled isopropanol to the extraction solution.
• Mix the tube three to five times using a vortex mixer.
• Place the tube into a −80°C freezer for at least five minutes ^4^.
• Remove the tube from the −80°C freezer and allow it to defrost.
• Centrifuge the tube for 10 minutes at full speed (ca. 10,625 g using a desktop centrifuge).
• Dry the pellet by gently pouring the supernatant away onto a tissue.
• Add 190 μl 70% EtOH and gently flick tube to mix the contents.
• Centrifuge the tube for two minutes at full speed (ca. 10,625 g using a desktop centrifuge).
• Dry the resulting pellet by removing the excess liquid using a P200 pipette set to 200 μl. Incubate the tube in a heat block set at 55°C for 5-10 minutes until it is fully dry.
• Pipette 30 μl dH _2_O into the tube to resuspend the DNA. This can be achieved by aspirating the liquid into a pipette tip and releasing is again several times until the pellet is no longer visible. Incubate the tube in a dry heat block for five to ten minutes at 65°C. The DNA can now be stored at 4°C (for long term use in applications other than genotyping), or immediately used in a PCR reaction.

## Results

### Characterisation studies

In previous studies from our lab we compared the concentration of DNA collected by skin swabbing and fin clipping, and the ability to amplify genes when using both techniques in stickleback and zebrafish. We also investigated the possibility for cross contamination of mucus samples, and changes to stress axis activity and behaviour following DNA collection. The data presented have all be published previously in Breacker *et al.* (2017) and Tilley *et al.* (2020).

*Concentration of DNA collected by fin clipping vs skin swabbing*

**Figure 1.**
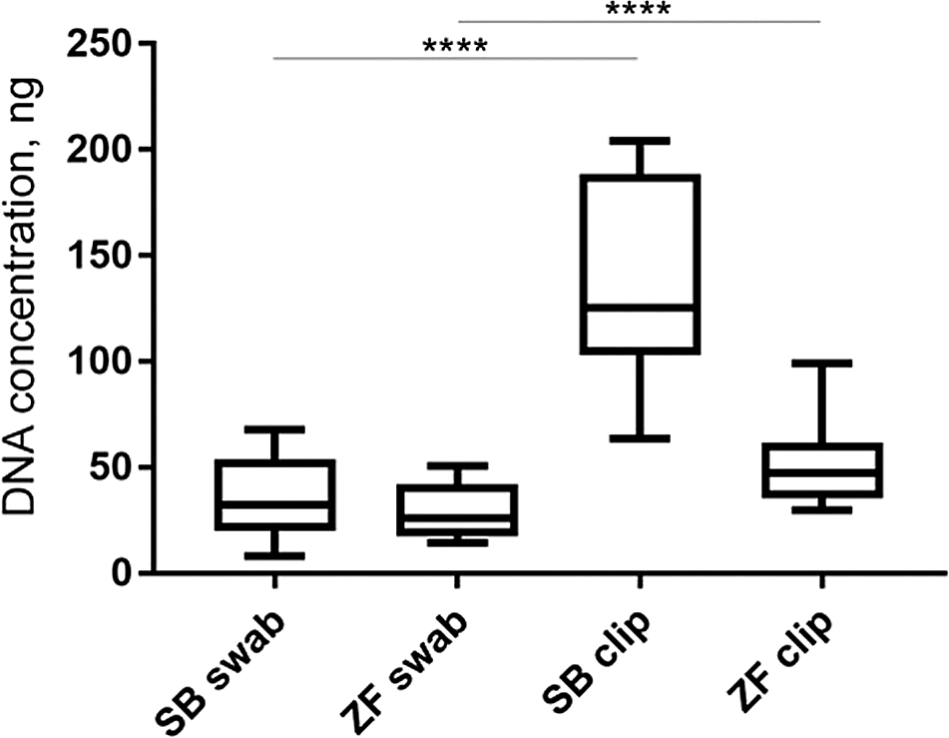
Graph of data from Table 1 comparing DNA concentration collected by skin swabbing or fin clipping either zebrafish or sticklebacks. This data is taken from Breacker *et al*. (2017). Fin clipping led to more DNA being collected in both species compared to skin swabbing. The same fish were used in both procedures. n = 10 zebrafish and n = 10 sticklebacks. One-way ANOVA followed by Tukey’s post hoc test. **** = p < 0.001. SB = stickleback and ZF = zebrafish.

Skin swabbing may be expected to be less invasive than fin clipping since it does not require removal of a portion of tissue from the animal. However, it was not clear whether both techniques are equally useful to genotype animals. We first investigated whether skin swabbing led to similar levels of DNA collection as fin clipping, the current standard procedure ( Xing *et al.*, 2014). Comparing swabs and fin clips taken from the same fish revealed that fin clips produced higher yields of DNA, suggesting that it is a more efficient method ( Table 1). However, the DNA samples collected using both methods had similar levels of purity, measured by calculating the 260/280 and 260/230 ratios using a spectrophotometer ( Table 1 and Figure 1). Pure DNA samples should have a 260/280 ratio of ~1.8 and a 260/230 ratio of between 2.0 and 2.2. However, slight deviations from this ratio may not affect amplification of genes by PCR.

**Table 1.** Comparison of DNA concentration from fin clips and skin swabs of the same sticklebacks and zebrafish. Skin swabbing led to a lower concentration of DNA extraction than fin clipping in both species. The quality of the DNA collected was similar when using both techniques. DNA concentration was measured using a spectrophotometer following extraction as described above. The purity of the DNA was measured using the 260/280 and 260/230 ratios. Pure DNA samples should have a 260/280 ratio of ~1.8 and a 260/230 ratio of between 2.0 and 2.2. We compared n = 10 sticklebacks and n = 10 zebrafish. Modified with permission from Breacker *et al*. (2017). Detailed methods for fin clipping, and a description of the experimental design can be found in Breacker *et al*. (2017) as well.

Species	DNA ng/nl	260/280 ratio	260/230 ratio	DNA ng/nl	260/280 ratio	260/230 ratio
Stickleback 1	61.4	2.09	1.53	204.1	2.1	1.99
Stickleback 2	23.3	2.01	1.89	189.3	2.12	1.61
Stickleback 3	8.3	2.12	1.4	122.4	2.06	1.42
Stickleback 4	51.2	2.11	1.26	188.5	2.08	1.67
Stickleback 5	31.2	2.05	2.01	143.8	2.08	1.41
Stickleback 6	26.3	2.08	1.92	75	2.06	1.63
Stickleback 7	67.9	2.04	1.63	112.4	2.02	1.81
Stickleback 8	45.4	2.02	1.79	112.9	2.11	1.41
Stickleback 9	33.3	2.05	1.06	128.4	2.09	1.86
Stickleback 10	10.2	2.064	1.97	63.7	2.1	1.94
**Mean stickleback**	35.85	2.064	1.646	134.05	2.082	1.675
Zebrafish 1	21.3	2.06	1.3	45.3	1.94	1.46
Zebrafish 2	17	2.01	2	35.6	1.97	1.4
Zebrafish 3	30.6	2.32	1.14	53.4	1.88	1.4
Zebrafish 4	23.3	1.41	0.42	49	1.99	1.93
Zebrafish 5	14.2	1.8	0.44	29.8	1.99	1.99
Zebrafish 6	28.9	1.95	2.46	44.4	1.97	1.77
Zebrafish 7	50.5	2.02	1.92	99.1	2.02	2.01
Zebrafish 8	41.4	1.95	1.04	87.2	1.97	1.77
Zebrafish 9	17.4	1.95	1.54	35.3	1.88	1.4
Zebrafish 10	43.4	1.95	1.04	49.4	2.21	1.53
**Mean zebrafish**	28.8	1.942	1.33	52.85	1.982	1.666

*PCR results comparing skin swabbing and fin clipping*

**Figure 2.**
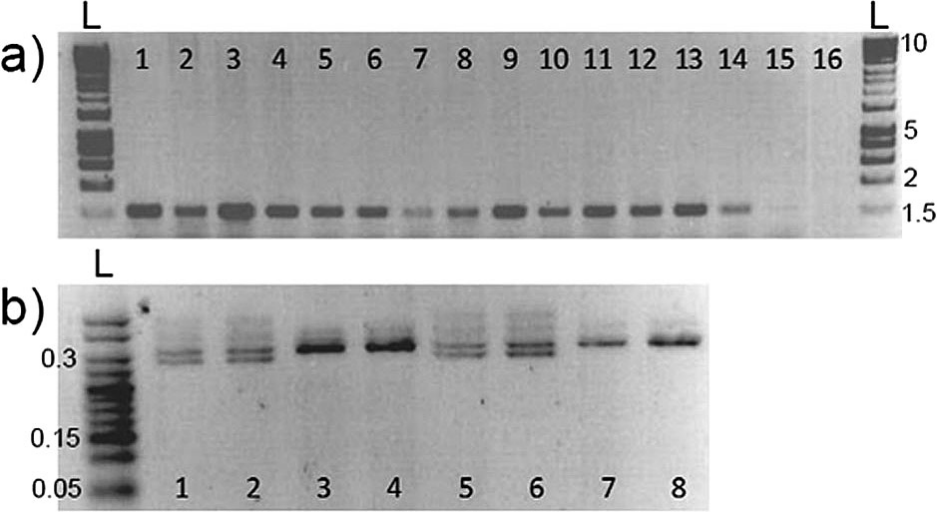
Representative PCR results from DNA samples. (a) Amplification of *mitfa* from zebrafish fin clips, swabs and size range; 1-3 = fin clips, standard length (measured from nose to beginning of tail fin) adults, at 6 months old; 4-6 = swabs standard length adults, at 6 months old; 7-9 = swabs from 20 mm standard length adults; 10-12 = swabs from 35 mm standard length adults; 13-15 = swabs from 50 mm standard length adults; 16 = blank. n = 15 zebrafish, one fish per band. The amplified band has a size of around 1,500 bp. (b) Amplification of *Isocitrate dehydrogenase* from stickleback swabs and size; 1-2 = 50 mm standard length adult male swabs; 3-4 = 50 mm standard length adult female swabs; 5-6 = 20 mm standard length adult male swabs; 7-8 = 20 mm standard length adult female swabs. n = 8 sticklebacks, one fish per band. Females produce a single 300 bp band, whereas male fish produce two bands that are 270 bp and 300 bp. L marks the position of the 1 Kb DNA ladders, and the numbers on the right-hand side show ladder band sizes in Kb. Reproduced with permission from Breacker *et al*. (2017).

DNA extracted from fin clipping is commonly used to identify animals via PCR amplification of marker genes. We investigated whether skin swabbing collected enough DNA to allow PCR amplification of genes in stickleback and zebrafish, despite the lower yield recovered compared to fin clipping. In this experiment we used a different group of zebrafish and sticklebacks from those presented in Table 1. In stickleback, we amplified the gene coding for the sex-linked marker *Isocitrate dehydrogenase* ( *IDH*). In zebrafish, we compared amplification of the *microphthalmia-associated transcription factor a* ( *mitfa*) gene using both sampling techniques. We also compared fish of different sizes, to show that swabbing can be used in animals that have not yet reach adulthood. In both zebrafish and stickleback, skin swabbing and fin clipping led to amplification of genes by PCR, suggesting that both techniques can be used for genetic identification. In addition, we were able to collect mucus from fish with a body size greater than 20 mm without damaging the animals ( Figure 2).

### Validation studies

Skin swabbing and fin clipping appear to be equally useful when amplifying genes to identify fish. We next examined whether mucus cross contamination occurs in fish held at high density, and whether either technique alters stress axis activation and behaviour following DNA collection.

*No cross contamination of mucus samples when fish are held at high density*

**Figure 3.**
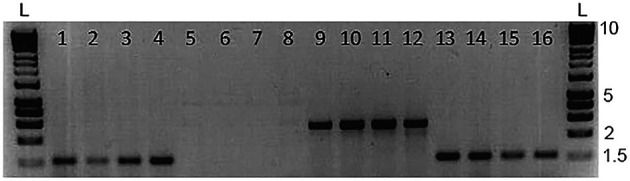
Representative data from a zebrafish cross-contamination test; 1-4 = WT DNA with *mitfa* primers; 5-8 = WT DNA with *gfp* primers; 9-12 = Vmat DNA with *gfp* primers; 13-16 = Vmat DNA with *mitfa* primers. We used n = 15 zebrafish in this experiment, and show n = 4 WT and n = 4 *Tg* ( *vmat2: GFP*) zebrafish here for clarity. The *mitfa* bands are approximately 1,500 bp long, and the *vmat* band is approximately 2,500 bp long. L marks the position of the 25 bp DNA ladders. Reproduced with permission from Breacker *et al*. (2017). Refer to Breacker *et al*. (2017) for detailed methods.

Skin swabbing appears to be a suitable technique to collect DNA from small laboratory fish without the need to excise fin tissue ( Tilley *et al.*, 2020; Breacker *et al*., 2017). We investigated the potential for cross contamination of mucus samples when housing zebrafish in close proximity in a small aquarium. We created a group by mixing 10 AB wild-type and 10 *Tg (vmat2:GFP)* zebrafish and maintained them in a small 3 L tank overnight. *Tg (vmat2: GFP)* carry a green fluorescent protein (GFP) transgene that can be amplified by PCR (Wen *et al*., 2008) and have long ornamental fins allowing them to be distinguished visually from the AB wild-type animals. We first swabbed the AB wild-types and then the *Tg (vmat2: GFP)* fish, and we did not assess whether this order of collecting DNA might influence the results. We amplified both *mitfa* (a control gene that should be present in all animals) and the gene coding for GFP, which should be present in the transgenic carriers but not wild-type. Figure 3 shows a subset of four of these fish for clarity. As hypothesised, *mitfa* was present in both genotypes ( Figure 3; lanes 1-4 AB and 13-16 *Tg (vmat2: GFP)*), whereas *gfp* was only present in the transgenic fish ( Figure 3; lanes 5-8 AB and 9-12 *Tg (vmat2: GFP)*). This suggests that cross-contamination of mucus had not occurred when keeping fish at high density.

*No changes in cortisol release after skin swabbing*

**Figure 4.**
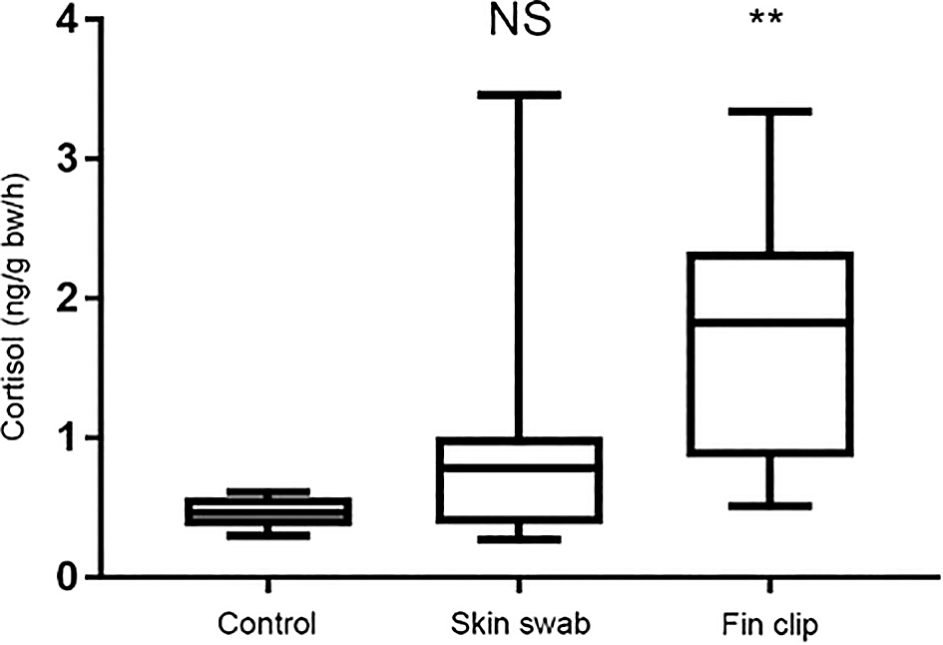
Changes in cortisol excretion in zebrafish following skin swabbing without MS-222 (tricaine) treatment or fin clipping following immersion in MS-222. We measured the concentration of cortisol release (in ng) per fish body weight (grams body weight) in an hour. n = 27 fish in each group. Kruskal-Wallis test followed by Dunn’s multiple comparisons test. ** indicates a significant difference compared to control, p < 0.001. NS indicates a non-significant difference compared to control. Reproduced with permission from Tilley *et al*. (2020). Detailed methods are available in Tilley *et al*. (2020), and the groups shown here have been taken from a larger experiment.

Release of cortisol – the primary stress hormone in vertebrates – can be used as a read-out of stress axis activity following experimental manipulation ( Sebire *et al*., 2007). We compared cortisol release in separate groups of sticklebacks and zebrafish that were either non-manipulated, fin clipped or swabbed. We then collected the cortisol they had excreted into their tank water one hour later as described previously ( Sebire *et al*., 2007, 2009; Ellis *et al.*, 2004). The cortisol was extracted from the fish’s holding water and quantified by radioimmunoassay. Collecting mucus by skin swabbing did not trigger an increase in cortisol release compared to control, non-manipulated fish that had not been handled at all, whereas fin clipping led to heightened release of this hormone ( Figure 4). This suggests that fin clipping is more stressful for fish than skin swabbing when used to sample DNA. This likely occurs because the fin clipping procedure involves application of the anaesthetic MS-222, whereas skin swabbing does not.

*Greater variability in results after clipping compared to swabbing*

We next examined the effect of skin swabbing and fin clipping on subsequent experimental data. We recorded the opercular beat rate (OBR, a read out of ventilation response to stress in fish ( Bell *et al.*, 2010; Brown *et al.*, 2005), and behaviour in the open field (Norton and Carreno Gutierrez, 2019), novel tank and black and white tests (for anxiety-like behaviour: Norton and Carreno Gutierrez, 2019; Blaser and Rosemberg, 2012; Egan *et al.*, 2009). Both skin swabbing and fin clipping caused complex changes to behaviour that varied over time ( Tilley *et al.*, 2020). Fin clipping caused a decrease in opercular beat rate in sticklebacks and an increase in zebrafish, whereas skin swabbing did not affect this behaviour. In the novel tank test, skin swabbing decreased the distance swum and increased time spent at the bottom of the tank by zebrafish, an index of anxiety-like behaviour on days 2 and 7. Conversely, fin clipping increased the distance swum in a novel tank by sticklebacks on day 1, and decreased zebrafish swimming on day 1 in both the novel tank and open field tests (see figure 7 in Tilley *et al.*, 2020). Fin clipping also led to greater variation in the data collected following DNA sampling compared to skin swabbing. Behavioural data collected subsequent to fin clipping showed more variable data compared to skin swabbed animals, meaning that more individuals need to be measured to test hypotheses with sufficient statistical power ( Tilley *et al.*, 2020). This means that collecting DNA by skin swabbing may lead to a reduction of total number of animals needed in experiments.

## Discussion

This protocol describes in detail the steps needed to collect DNA from small-bodied fish species using skin swabbing. We have also included findings from our previous research, comparing PCR amplification of genes in zebrafish and stickleback, and investigating the potential for cross-contamination of mucus samples during husbandry ( Tilley *et al.*, 2020; Breacker *et al.*, 2017). We also show that skin swabbing does not activate release of the stress hormone cortisol, suggesting that it may be less stressful than fin clipping ( Tilley *et al.*, 2020). In summary, both skin swabbing and fin clipping can alter stress axis activation and behaviour. However, skin swabbing appeared to have less impact upon zebrafish and stickleback than fin clipping, since fewer of the read-outs that we measured were altered by this technique.

Several previous studies have shown that fin clipping can have side effects that may alter the outcome of experimental studies. For example, it can lead to secondary infections and elevate the non-specific immune response ( Dash *et al.*, 2018). In addition, fin clipping is painful for fish ( Deakin *et al.*, 2019), and therefore requires them to be anaesthetised, raising the potential for further disruption of the stress response or behaviour. As a result of these observations, concerns have been raised about the welfare implications of collecting DNA by fin clipping.

Skin swabbing appears to be a less invasive method for obtaining DNA samples since it only requires a small amount of mucus to be removed from the fish’s flank. In a recent set of experiments, we demonstrated that skin swabbing can be performed in the absence of anaesthetic without exacerbating changes in cortisol release ( Tilley *et al.*, 2020). However, our previous comparison of both techniques uncovered complex changes in gene expression and behaviour that varied over time following DNA collection. Importantly, we observed a greater variation in behaviour in groups of fish that had been fin clipped compared to those that had been swabbed ( Tilley *et al*., 2020), meaning that fewer animals need to be measured in order to generate sufficiently powered data when DNA is collected with a swab. This can improve the quality of data collection and reduce the number of animals used, thereby decreasing the cost of experiments.

Removal of tissue during fin clipping led to an acute increase in water-borne cortisol release in both fish species whereas skin swabbing did not ( Tilley *et al.*, 2020). Most changes to experimental data due to fin clipping appeared on the first day after DNA collection. In contrast, the changes in gene expression and behaviour caused by skin swabbing tended to appear between two and seven days after manipulation, perhaps due to the activation of an immune response, or changes in ionic or osmotic regulation ( Dash *et al.*, 2018). However, we also observed changes in the behaviour of control non-manipulated fish over time, demonstrating the difficulty of comparing repeat measures of behaviour and accounting for the influence of routine maintenance on animals ( Tilley *et al.*, 2020). Some differences between species were observed as well. Zebrafish displayed more changes in behaviour than sticklebacks after manipulation; and both fin clipping and skin swabbing had a greater effect on gene expression in stickleback than zebrafish ( Tilley *et al.,* 2020).

We also confirmed that cross-contamination of mucus samples did not occur when fish were held at high densities overnight. We maintained a group of wild-type and transgenic zebrafish at densities greater than those recommended for normal housing conditions ( Le Vin *et al.*, 2011). Nevertheless, we were still unable to amplify the gene coding for Green fluorescent protein in wild-type fish, despite crowding them together with transgenic *Tg (vmat2:GFP)* carriers ( Figure 2). This suggests that skin swabbing is an appropriate technique to collect DNA even when fish have been in close contact with each other.

In summary, our previous research has shown that skin swabbing can yield DNA concentrations and purities that are comparable to fin clipping when combined with a low-cost recovery method ( Table 1, adapted from Breacker *et al*., 2017). Swabbing is a suitable technique for small laboratory fish that are as small as 20 mm – when used on smaller animals there is the potential for harm when swabbing ( Breacker *et al.*, 2017). Skin swabbing triggers fewer changes in stress axis activation, behaviour and gene expression than fin clipping. In addition, no sharp scalpels are needed when collecting DNA by skin swabbing, therefore reducing the possibility of harm to researchers. Skin swabbing is simple to perform once researchers have been trained in the technique, although care must be taken to swab the fish from anterior to posterior using very light pressure. In the future, the use of skin swabbing might be expanded to other species that have a mucus layer on their body such as fish, frogs and toads.

## Data availability

### Underlying data

Open Science Framework: Skin swabbing protocol to collect DNA samples from small-bodied fish species, https://doi.org/10.17605/OSF.IO/HS83T/.

This project contains the following underlying data:

- Breacker *et al.* (2017) raw data.xlsx
- Skin swabbing protocol to collect DNA samples from small-bodied fish species file 2.csv
- Tilley *et al.* (2020) raw data.xlsx

### Extended data

Figshare: Swabbing videos 1 and 2, https://doi.org/10.6084/m9.figshare.17049590 ( Norton *et al.*, 2021).

This project contains the following extended data:

- Swabbing video 1.mp4
- Swabbing video 2.mp4

Data are available under the terms of the Creative Commons Attribution 4.0 International license (CC-BY 4.0).

## Reporting guidelines

Open Science Framework: ARRIVE checklist for Tilley *et al.*, Skin swabbing protocol to collect DNA samples from small-bodied fish species, https://doi.org/10.17605/OSF.IO/HS83T/.

Data are available under the terms of the Creative Commons Attribution 4.0 International license (CC-BY 4.0).

## Footnotes

^1^
We have posted a swab in DNA extraction solution to ourselves using the normal postal service. DNA was recovered successfully from the swab one week later.

^2^
This protocol can be completed in around four hours – one hour to extract DNA, two hours to run a PCR and one hour to visualise the product on an electrophoresis gel. We routinely identify around twenty animals at a time.

^3^
Other research groups have reported DNA extraction by the hot-shot method following skin swabbing: e.g. Venta *et al*. (2020).

^4^
The tubes can be stored at -80°C overnight if required.
