# Supplementary material for: Skin swabbing protocol to collect DNA samples from small-bodied fish species
Source: F1000Res. 2024 Jun 27;10:1064. Originally published 2021 Oct 19. [Version 2] doi: 10.12688/f1000research.73115.2 (PMC11342036; doi:10.12688/f1000research.73115.2)
Supplement: Peer review history

# Reviewer report: Reviewer response for version 2

**DOI:** 10.5256/f1000research.165869.r296346

**Published:** 2024-08-22

**Authors:** Luigi Margiotta-Casaluci

**Affiliations:** 1 Kings College London, London, UK

The authors have satisfactorily addressed all the comments and concerns that I shared in the review of Version 1.

Is the rationale for developing the new method (or application) clearly explained?

Partly

Are the 3Rs implications of the work described accurately?

Partly

Is the description of the method technically sound?

Yes

If applicable, is the statistical analysis and its interpretation appropriate?

Partly

Are the conclusions about the method and its performance adequately supported by the findings presented in the article?

Partly

If any results are presented, are all the source data underlying the results available to ensure full reproducibility?

Yes

Are a suitable application and appropriate end-users identified?

Yes

Are sufficient details provided to allow replication of the method development and its use by others?

Yes

Reviewer Expertise:

Fish physiology, endocrinology, behaviour, 3Rs, toxicology. Referee suggested by the NC3Rs for their scientific expertise and experience in assessing 3Rs impact.

I confirm that I have read this submission and believe that I have an appropriate level of expertise to confirm that it is of an acceptable scientific standard.

# Reviewer report: Reviewer response for version 2

**DOI:** 10.5256/f1000research.165869.r296345

**Published:** 2024-06-28

**Authors:** Errol D Cason

**Affiliations:** 1 University of the Free State, Bloemfontein, South Africa

no further comments

Is the rationale for developing the new method (or application) clearly explained?

Yes

Are the 3Rs implications of the work described accurately?

Yes

Is the description of the method technically sound?

Yes

If applicable, is the statistical analysis and its interpretation appropriate?

Yes

Are the conclusions about the method and its performance adequately supported by the findings presented in the article?

Yes

If any results are presented, are all the source data underlying the results available to ensure full reproducibility?

Yes

Are a suitable application and appropriate end-users identified?

Yes

Are sufficient details provided to allow replication of the method development and its use by others?

Yes

Reviewer Expertise:

Genetics, Genomics, Microbiomes,

I confirm that I have read this submission and believe that I have an appropriate level of expertise to confirm that it is of an acceptable scientific standard.

# Reviewer report: Reviewer response for version 1

**DOI:** 10.5256/f1000research.76741.r144865

**Published:** 2022-08-17

**Authors:** Errol D Cason

**Affiliations:** 1 University of the Free State, Bloemfontein, South Africa

This paper provides detailed steps to collect DNA from small-bodied fish species using skin swabbing. This is offered as a alternative, easier, less invasive and more ethical methods of acquiring DNA from fish during trials when compared to the standard method of fin clipping.

 Evidence is provided that enough DNA an be obtained for downstream application with less stress, discomfort and pain to the fish.

 In essence this paper is a "manual" for methods already put out in Breaker *et al. *(2017) and Tilley *et al.* (2020). ^1^^,^^2^ It appears to only exist as a easily accessible and referenceable recipe. It will save scientists time and effort to go and dig through the other papers if all they really want is A) to know if the method works and B) how to do it.

 While this is not a novel study, and is just a bullet point + video version of previously published methods, I do however feel that this is not a bad thing. I can see how the other two papers might not be getting the traction they deserve for a method that mostly works just as well but you don't need to mutilate an animal to get DNA. I therefore see no reason not to index this.

Is the rationale for developing the new method (or application) clearly explained?

Yes

Are the 3Rs implications of the work described accurately?

Yes

Is the description of the method technically sound?

Yes

If applicable, is the statistical analysis and its interpretation appropriate?

Yes

Are the conclusions about the method and its performance adequately supported by the findings presented in the article?

Yes

If any results are presented, are all the source data underlying the results available to ensure full reproducibility?

Yes

Are a suitable application and appropriate end-users identified?

Yes

Are sufficient details provided to allow replication of the method development and its use by others?

Yes

Reviewer Expertise:

Genetics, Genomics, Microbiomes,

I confirm that I have read this submission and believe that I have an appropriate level of expertise to confirm that it is of an acceptable scientific standard.

## References

[1] : A Low-Cost Method of Skin Swabbing for the Collection of DNA Samples from Small Laboratory Fish. *Zebrafish* .14(1) : 10.1089/zeb.2016.1348 35-41 10.1089/zeb.2016.1348 27788059 PMC5312459

[2] : Skin swabbing is a refined technique to collect DNA from model fish species. *Scientific Reports* .2020;10(1) : 10.1038/s41598-020-75304-1 10.1038/s41598-020-75304-1 33097784 PMC7584585

# Reviewer report: Reviewer response for version 1

**DOI:** 10.5256/f1000research.76741.r117243

**Published:** 2022-01-27

**Authors:** Luigi Margiotta-Casaluci

**Affiliations:** 1 Kings College London, London, UK

**General comments**

 In this article, the authors describe a skin swabbing protocol for DNA collection from small laboratory fish species that could provide a suitable alternative to the commonly used fin clipping procedure. The latter is an increasingly common procedure used in most zebrafish laboratories at global level. As mentioned by the authors, 85% of zebrafish laboratories use fin clipping to collect DNA. This procedure requires anaesthesia and involve surgical removal of tissue; therefore, the development of alternative minimally invasive methods in this area may have significant implications for the welfare of laboratory fish. The alternative skin swabbing protocol presented in this article is well described, and the work provides a valuable contribution to the advancement of laboratory fish welfare. However - although the potential welfare benefits of skin swabbing vs fin clipping may sound obvious - I believe that the specific datasets presented in the article do not allow a robust cost-benefit assessment of the protocol from a 3Rs perspective and the evaluation of its superiority compared to the commonly used fin clipping procedure. Hence, I suggest that that aspect of the article should be improved, expanded, and integrated with other data (if available). Specific comments and suggestions are provided below.

**Skin swabbing protocol**

 The methodology described in the article appears to be sound, and I believe that the details provided allow to reliably replicate the simple method in other laboratories. The supplemental video clips are clear and very informative.

 Despite a lower DNA yield obtained with skin swabs, the DNA samples collected using both methods had similar levels of purity. The authors demonstrated that DNA extracted from skin mucus allows a reliable amplification of target genes, and that no mucus cross contamination occurs in fish held at high density.

 The supplemental videos are very good and clearly demonstrate the key practical steps. In the video the operator apparently restrains the fish on the sponge by gently covering the eyes with a gloved finger. I suppose this procedure calms the fish and reduces potential harm. Do the authors suggest that this should be a key step of the protocol? If yes, I suggest to add more details about this step in the main text.

 In the published protocol the authors reported that fish were immersed in lidocaine for 45 minutes before performing the procedure. Can the authors explain why this duration was selected? Does water temperature affect uptake and pharmacokinetics of lidocaine in different species, and in turn the incubation time? Considering the time needed to perform the skin swab, how many fish should be added to the lidocaine solution to ensure that no fish experiences an excessive exposure? Considering the use of static water conditions for the exposure to lidocaine, I foresee that the temperature may change very rapidly unless the room temperature is controlled. Can the authors recommend a specific strategy to ensure the maintenance of optimal water temperature (and hence the reduction of stress) throughout the lidocaine exposure phase? E.g. using an incubator or a dedicated genotyping rack.

 Table 1 shows the comparison of DNA concentration from fin clips and skin swabs of the same sticklebacks and zebrafish. I believe the top row displaying the column titles is missing.

**Cross-contamination**

 To evaluate the potential cross contamination of mucus samples when fish are held at high density, the authors kept 10 AB wild-type and 10 transgenic zebrafish in a small 3 L tank overnight. The analysis show that, in these conditions, there is no contamination. However, it appears that the authors reached this conclusion after a non-random sampling where all ABs were swabbed first, followed by all transgenic fish (displaying a longer tail). Although one may conclude that there is no cross contamination in the fish tank, the experimental design does not allow to evaluate whether the full procedure itself (i.e. including the transfer onto the sponge) may be a source of contamination. Although the probability is low, do the authors think that the sponge may represent a contamination source? If yes, do they recommend to replace the sponge after sampling a certain number of fish (e.g. how many?).

 Similar considerations are valid for gloves and nets. How often should these elements be replaced/washed?

**Claims of adversity of fin clipping – Introduction and discussion**

 The Introduction reports the following statement: “ *However, despite its popularity,* *there is evidence that fin clipping can alter health and welfare, leading to infection and altering growth and survival (Taslima et al., 2015)*”. However, Taslima et al. (2015) ^1^ appears to be an aquaculture-related paper focused on Nile tilapia and, as far as I can see, that paper does not provide any evidence of fin clipping-induced damage. Taslima et al. (2015) provided the following generic statement “ *tissue biopsy may have negative impacts on fish, potentially including infection and effects on survival, growth or behaviour”,* and to support this statement they cited Le Vin et al. (2011) (this paper is also cited in the present manuscript).[red-2] The latter concerns the validation of swabs methods for DNA sampling in fish. Again, the study did not investigate the adverse effects of fin clipping. On the other hand, Le Vin et al. (2011) also stated that fin clipping can be detrimental. This time, to support that statement, they cited several papers from the 60s, 70s, and 80s. However, those papers appear to be referred mainly to aquaculture and field ecology studies where fish are reintroduced in the environment after fin-clipping. Going back to the introduction of the present paper, I believe that whereas the potential adverse effects of fin clipping on behaviour are evidenced with appropriate supporting citations, the other potential adverse effects on infection, growth. and survival are not.

 Is there any evidence that fin clipping can adversely affect growth, survival, and infection susceptibility in a laboratory setting? If not, the authors should specific that it is not the case, and discuss potential effects observed in aquaculture studies providing an accurate citation and reporting of the primary papers. If yes, it would be useful to have specific data on this important aspect.

 Similarly, in the Discussion, the authors wrote that “ *Several previous studies have shown that fin clipping can have side effects that may alter the outcome of experimental studies. For example, it can lead to secondary infections and elevate the non-specific immune response (Dash et al., 2018)*”. However, Dash et al. (2018) do not seem to mention fin clipping at all, as it is a study focused on the role played by fish mucus in health maintenance. ^3^ Please, revise this reference and replace with primary papers, if possible.

**Behavioural studies and variability of behavioural and stress endpoints**

 In the present paper, the negative effects of fin clipping of fish behaviour was used as key argument to justify the potential superiority of skin swabbing over fin clipping. However, I believe that this argument is only weakly supported by the data presented here. I suggest to add more data, if available, to strengthen this argument. 

 The methodological description of the behavioural tests is too basic and requires more details. For example, in the novel tank diving test, fish were observed/recorded for the first five minutes. Is this observation time justified by other studies for both stickleback and zebrafish? If yes, what are the supporting references? Were multiple fish/tanks filmed simultaneously using multiple tanks? If yes, did the operator add the fish using a specific sequence or removed the first seconds of the video where the operator was still in the room? (The operator in the room is a source of stress). The results section indicates that multiple time points were tested. However, the different timepoints and the rationale behind their selection are not described in the methods section. The importance of providing an accurate description of the methods is clearly demonstrated by the following, and more important, point.

 The results section indicates that there is “ *Greater variability in results after clipping compared to swabbing*”. I found that statement confusing as, by itself, it leads one to believe that there is a variability in genotyping results whereas the authors refer to behavioural and stress endpoints (which are not the focus of the paper). Later, the article provides the following statement: “ *Behavioural data collected subsequent to fin clipping showed more variable data compared to skin swabbed animals, meaning that more individuals need to be measured to test hypotheses with sufficient statistical power (Tilley et al., 2020). ^4^ This means that collecting DNA by skin swabbing may lead to a reduction of total number of animals needed in experiments*”. In my opinion, it is unclear why skin swabbing could allow to use fewer animals. Do the authors refer to a specific context? I suggest to clarify this point or to remove it.

 Overall, this specific aspect of the work (i.e. data variability) has been highlighted very strongly in the manuscript; however, my main concern is the data does not appear to support the hypothesis of a treatment-dependent effect. The present manuscript does not provide any figure or table of the behavioural data; however, this data can be retrieved from Tilley et al. (2020). Reading the latter, it is possible to note that the observed behavioural effects appeared to be (almost always) driven by fish handing procedures and not by fin clipping or skin swabbing themselves. The same consideration is valid for the assessment of opercular beats. The fact that control fish display behavioural responses more often (or as often) as fish that underwent DNA collection weakens the argument of treatment-specific effects, and hence treatment-specific benefits, including 3Rs benefits. Can the authors provide more data concerning this aspect?

**Effects on cortisol release**

 To measure the effects of the different procedures on the stress axis, the authors measured cortisol excretion in the tanks of fish that underwent 1) no manipulation, 2) skin swabbing, 3) fin clipping. I believe that the experimental design described in the article is not suitable to provide an answer to the specific research question considered here. Although measuring the cortisol released by a non-manipulated group provided a useful reference value, I believe that there are multiple factors to consider for a more rigorous comparison. 1) Fish handing – it is known that fish netting and air contact are major stress factors that induce an increase of cortisol release in the surrounding water (Ellis, James, Scott 2004). ^5^ Measuring cortisol concentrations in tanks of non-manipulated fish will very likely indicate lower values, independently from skin swabbing or fin clipping. Hence, a more appropriate control would be represented by fish that underwent the same handling (i.e. netting and transfer to a new tank) without DNA sampling procedure. 2) The pre-procedure treatment of fish that underwent skin swabbing or fin clipping were very different (i.e. 45 minutes exposure to lidocaine in a confined space versus rapid treatment with MS-222). From the results presented here, it is not possible to evaluate whether the 45 minutes pre-treatment with lidocaine did or did not affect cortisol release. In conclusion, this suggests that an experiment aimed at evaluating the stress response to different DNA sampling procedures should include specific negative controls for each treatment type and fish handling procedure.

**Other comments**

 In the discussion, the authors reported that “ *changes in gene expression and behaviour caused by skin swabbing tended to appear between two and seven days after manipulation, perhaps due to the activation of an immune response, or changes in ionic or osmotic regulation (Dash et al., 2018*)’. In my opinion, this possibility is concerning and should be explored in further studies, as it may hamper any 3Rs benefit of skin swabs. For example, are swabbed fish in the laboratory more susceptible to infection and disease in the longer term compared to fin clipped fish?

 The reference de Lombaert et al. (2017) is not present in the reference list.

Is the rationale for developing the new method (or application) clearly explained?

Partly

Are the 3Rs implications of the work described accurately?

Partly

Is the description of the method technically sound?

Yes

If applicable, is the statistical analysis and its interpretation appropriate?

Partly

Are the conclusions about the method and its performance adequately supported by the findings presented in the article?

Partly

If any results are presented, are all the source data underlying the results available to ensure full reproducibility?

Yes

Are a suitable application and appropriate end-users identified?

Yes

Are sufficient details provided to allow replication of the method development and its use by others?

Yes

Reviewer Expertise:

Fish physiology, endocrinology, behaviour, 3Rs, toxicology. Referee suggested by the NC3Rs for their scientific expertise and experience in assessing 3Rs impact.

I confirm that I have read this submission and believe that I have an appropriate level of expertise to confirm that it is of an acceptable scientific standard, however I have significant reservations, as outlined above.

## References

[1] : DNA sampling from mucus in the Nile tilapia, Oreochromis niloticus: minimally invasive sampling for aquaculture-related genetics research dust. *Aquac. Res* .2015;47(12) :

[2] : Validation of swabs as a non-destructive and relatively non-invasive DNA sampling method in fish. *Mol Ecol Resour* .2011;11(1) : 10.1111/j.1755-0998.2010.02909.x 107-9 10.1111/j.1755-0998.2010.02909.x 21429106

[3] : Epidermal mucus, a major determinant in fish health: a review. *Iran J Vet Res* .2018;19(2) :72-81 30046316 PMC6056142

[4] : Swabbing videos 1 and 2. *f1000research.com Media* .2021;

[5] : A non-invasive stress assay based upon measurement of free cortisol released into the water by rainbow trout. *Journal of Fish Biology* .2004;65(5) : 10.1111/j.0022-1112.2004.00499.x 1233-1252 10.1111/j.0022-1112.2004.00499.x

# Response

**Published:** 2024-04-30

**Authors:** Will Norton

**Affiliations:** University of Leicester, UK

**Author Response:**

 The reviewer has provided us with excellent feedback upon the first version of this study. We have answered these comments in full, and our responses to the comments are included below. We are very sorry that it has taken us so long to respond to these suggestions, and we hope that you still accept to re-review this paper. A change in person circumstances prevented us from addressing your suggestions sooner. Thank you for reading this study so thoroughly, and for helping us to improve this manuscript.

***Reviewer Comment:***

*Although the potential welfare benefits of skin swabbing vs fin clipping may sound obvious, I believe that the specific datasets presented in the article do not allow a robust cost-benefit assessment of the protocol from a 3Rs perspective and the evaluation of its superiority compared to the commonly used fin clipping procedure. Hence, I suggest that that aspect of the article should be improved, expanded, and integrated with other data (if available). Specific comments and suggestions are provided below.*

 1. *The methodology described in the article appears to be sound, and I believe that the details provided allow to reliably replicate the simple method in other laboratories. The supplemental video clips are clear and very informative.*

**Author Response:**

 We thank the reviewer for this positive comment about our article. The video forms a core part of this protocol, and bearing this in mind we have now made a new, more professional, film demonstrating the swabbing technique. This video has been incorporated into the updated version of this paper.

***Reviewer Comment:***

 2. *Despite a lower DNA yield obtained with skin swabs, the DNA samples collected using both methods had similar levels of purity. The authors demonstrated that DNA extracted from skin mucus allows a reliable amplification of target genes, and that no mucus cross contamination occurs in fish held at high density*.

**Author Response:**

 Thank you for highlighting this aspect of our research.

***Reviewer Comment:***

 3. *The supplemental videos are very good and clearly demonstrate the key practical steps. In the video the operator apparently restrains the fish on the sponge by gently covering the eyes with a gloved finger. I suppose this procedure calms the fish and reduces potential harm. Do the authors suggest that this should be a key step of the protocol? If yes, I suggest to add more details about this step in the main text.*

**Author Response:**

 The reviewer is correct that gently covering the eyes of the fish can help stop them from moving. We routinely carry this out using a hand net. Our new video shows this step of the protocol in more detail. We have also added this information to the main text describing the technique as suggested.

***Reviewer Comment:***

 4. *In the published protocol the authors reported that fish were immersed in lidocaine for 45 minutes before performing the procedure. Can the authors explain why this duration was selected? Does water temperature affect uptake and pharmacokinetics of lidocaine in different species, and in turn the incubation time? Considering the time needed to perform the skin swab, how many fish should be added to the lidocaine solution to ensure that no fish experiences an excessive exposure?* *Considering the use of static water conditions for the exposure to lidocaine, I foresee that the temperature may change very rapidly unless the room temperature is controlled. Can the authors recommend a specific strategy to ensure the maintenance of optimal water temperature (and hence the reduction of stress) throughout the lidocaine exposure phase? E.g. using an incubator or a dedicated genotyping rack.*

**Author Response:**

 Since submitting the first version of this protocol, we have published a follow up study that shows that lidocaine treatment is not needed for DNA collection by skin swabbing (Tilley et al., 2023, Validating skin swabbing as a refined technique to collect DNA from small-bodied fish species). We have therefore removed all mentions of lidocaine pre-treatment from the protocol steps included here.

***Reviewer Comment:***

 5. *Table 1 shows the comparison of DNA concentration from fin clips and skin swabs of the same sticklebacks and zebrafish. I believe the top row displaying the column titles is missing.*

**Author Response:**

 Thank you for spotting this error. Table 1 does miss the top column indicating whether the samples came from swabbed or fin clipped fish. We have now replaced this table with a new version that contains this information.

***Reviewer Comment:***

 6. *To evaluate the potential cross contamination of mucus samples when fish are held at high density, the authors kept 10 AB wild-type and 10 transgenic zebrafish in a small 3 L tank overnight. The analysis show that, in these conditions, there is no contamination. However, it appears that the authors reached this conclusion after a non-random sampling where all ABs were swabbed first, followed by all transgenic fish (displaying a longer tail). Although one may conclude that there is no cross contamination in the fish tank, the experimental design does not allow to evaluate whether the full procedure itself (i.e. including the transfer onto the sponge) may be a source of contamination. Although the probability is low, do the authors think that the sponge may represent a contamination source? If yes, do they recommend to replace the sponge after sampling a certain number of fish (e.g. how many?).*

**Author Response:**

 The reviewer makes a good point about the possible transfer of mucus on the sponge leading to cross-contamination. We routinely rinse the sponge between sample collection, an important detail that was not described in our protocol. We have now added this information to the updated version of the protocol as suggested.

***Reviewer Comment:***

 7. *Similar considerations are valid for gloves and nets. How often should these elements be replaced/washed?*

**Author Response:**

 We routinely wash the net that is used to restrain the fish between sampling. This detail has been added to the protocol as per the reviewer’s suggestion. However, we do not change gloves, because we have not noticed any issues with cross-contamination, and this is our standard practice when handling animals and genotyping.

***Reviewer Comment:***

 8. *The Introduction reports the following statement: “However, despite its popularity, there is evidence that fin clipping can alter health and welfare, leading to infection and altering growth and survival (Taslima et al., 2015)”. However, Taslima et al. (2015) appears to be an aquaculture-related paper focused on Nile tilapia and, as far as I can see, that paper does not provide any evidence of fin clipping-induced damage. Taslima et al. (2015) provided the following generic statement “tissue biopsy may have negative impacts on fish, potentially including infection and effects on survival, growth or behaviour”, and to support this statement they cited Le Vin et al. (2011) (this paper is also cited in the present manuscript). The latter concerns the validation of swabs methods for DNA sampling in fish. Again, the study did not investigate the adverse effects of fin clipping. On the other hand, Le Vin et al. (2011) also stated that fin clipping can be detrimental. This time, to support that statement, they cited several papers from the 60s, 70s, and 80s. However, those papers appear to be referred mainly to aquaculture and field ecology studies where fish are reintroduced in the environment after fin-clipping. Going back to the introduction of the present paper, I believe that whereas the potential adverse effects of fin clipping on behaviour are evidenced with appropriate supporting citations, the other potential adverse effects on infection, growth. and survival are not. Is there any evidence that fin clipping can adversely affect growth, survival, and infection susceptibility in a laboratory setting? If not, the authors should specific that it is not the case, and discuss potential effects observed in aquaculture studies providing an accurate citation and reporting of the primary papers. If yes, it would be useful to have specific data on this important aspect.*

**Author Response:**

 The reviewer makes an important point here. Our statement about effects on growth, infection and survival are not sufficiently supported by the references that we have provided. This has occurred because we over-interpreted the Taslima et al. study, which in turn mis-cited earlier papers including Le Vin et al. 2011. We have now modified the introduction section to reflect this, deleting the suggestion that fin clipping can alter growth, survival and infection. We apologise for this mistake, and hope that the new version of the introduction is acceptable in its current format.

***Reviewer Comment:***

 9. *Similarly, in the Discussion, the authors wrote that “Several previous studies have shown that fin clipping can have side effects that may alter the outcome of experimental studies. For example, it can lead to secondary infections and elevate the non-specific immune response (Dash et al., 2018)”. However, Dash et al. (2018) do not seem to mention fin clipping at all, as it is a study focused on the role played by fish mucus in health maintenance. Please, revise this reference and replace with primary papers, if possible.*

**Author Response:**

 I am afraid that this is also a mistake on our part. The Dash et al. paper does indeed focus on mucus sampling and not fin clipping, making our statement incorrect. We have removed this sentence in the discussion as suggested by the reviewer. Although skin swabbing is a refined technique compared to fin clipping, it is important not to overstate its benefits. We thank the reviewer for spotting these errors and pointing them out to us.

***Reviewer Comment:***

 10. *In the present paper, the negative effects of fin clipping of fish behaviour was used as key argument to justify the potential superiority of skin swabbing over fin clipping. However, I believe that this argument is only weakly supported by the data presented here. I suggest to add more data, if available, to strengthen this argument.*

**Author Response:**

 The main aim of this paper is to provide an easy-to-follow protocol for researchers who wish to adopt skin swabbing for DNA collection. The experimental data that is shown aims to provide a background for the technique, and to convince researchers to give it a go. All of the data shown here is modified from previous publications (e.g., Tilley et al., 2020; Breacker et al., 2017). However, the reviewer is correct that this issue could be presented more clearly. To address this issue, we have now added a second data table, modified from Tilley et al., 2020, that shows the greater variability in gene expression and behavioural data following fin clipping compared to skin swabbing.

***Reviewer Comment:***

 11. *The methodological description of the behavioural tests is too basic and requires more details. For example, in the novel tank diving test, fish were observed/recorded for the first five minutes. Is this observation time justified by other studies for both stickleback and zebrafish? If yes, what are the supporting references? Were multiple fish/tanks filmed simultaneously using multiple tanks? If yes, did the operator add the fish using a specific sequence or removed the first seconds of the video where the operator was still in the room? (The operator in the room is a source of stress). The results section indicates that multiple time points were tested. However, the different timepoints and the rationale behind their selection are not described in the methods section. The importance of providing an accurate description of the methods is clearly demonstrated by the following, and more important, point.*

**Author Response:**

 As per the responses above, the behavioural information mentioned here is not the main focus of this paper. Rather, we have used data from Tilley et al., 2020 to demonstrate the variability in experimental results seen after fin clipping. The behaviour methods that we have used here are standard for the field of zebrafish research. The novel tank diving test protocol mentioned here is based upon the published work of Egan and colleagues in zebrafish, (Egan et al., 2009 – now included in the reference list), and modified for sticklebacks by Gutierrez and colleagues (Norton and Gutierrez, 2019). In brief, we recorded a single fish at a time for 5 minutes, and analysed the entirety of the behavioural response. Each timepoint that we looked at was recorded separately, using the same setup in the same room. The experimenter was present in the room throughout recording. 

***Reviewer Comment:***

 12. *The results section indicates that there is “Greater variability in results after clipping compared to swabbing”. I found that statement confusing as, by itself, it leads one to believe that there is a variability in genotyping results whereas the authors refer to behavioural and stress endpoints (which are not the focus of the paper). Later, the article provides the following statement: “Behavioural data collected subsequent to fin clipping showed more variable data compared to skin swabbed animals, meaning that more individuals need to be measured to test hypotheses with sufficient statistical power (Tilley et al., 2020). This means that collecting DNA by skin swabbing may lead to a reduction of total number of animals needed in experiments”. In my opinion, it is unclear why skin swabbing could allow to use fewer animals. Do the authors refer to a specific context? I suggest to clarify this point or to remove it.*

**Author Response:**

 We published data showing that compared to skin swabbing, fin clipping led to a greater variation in gene expression and behavioural data in Tilley et al., 2020. This data used an asymptotic test for the equality of coefficients of variation from k populations to contrast physiological and behavioural data after skin swabbing or fin clipping. When compared to control undisturbed fish, fin clipping led a large variation in data whereas skin swabbing did not. This means that fewer animals would need to be included in each experimental group in order to collect statistically significant data, if skin swabbing is used for DNA collection rather fin clipping. We have included table 2 in this manuscript, and re-written the results and discussion section to make this clearer.

***Reviewer Comment:***

 13. *Overall, this specific aspect of the work (i.e. data variability) has been highlighted very strongly in the manuscript; however, my main concern is the data does not appear to support the hypothesis of a treatment-dependent effect. The present manuscript does not provide any figure or table of the behavioural data; however, this data can be retrieved from Tilley et al. (2020). Reading the latter, it is possible to note that the observed behavioural effects appeared to be (almost always) driven by fish handing procedures and not by fin clipping or skin swabbing themselves. The same consideration is valid for the assessment of opercular beats. The fact that control fish display behavioural responses more often (or as often) as fish that underwent DNA collection weakens the argument of treatment-specific effects, and hence treatment-specific benefits, including 3Rs benefits. Can the authors provide more data concerning this aspect?*

**Author Response:**

 The data shown here is modified from Tilley et al., 2020, where we investigate which components of the fin clipping and swabbing procedures trigger a stress response in fish. While handling fish does trigger stress, we were able to show that the fin clipping procedure in its entirety is more stressful than the swabbing or handling alone. We have included a new table 2 in this version of the manuscript that shows some of this information, as suggested by the reviewer.

***Reviewer Comment:***

 14. *To measure the effects of the different procedures on the stress axis, the authors measured cortisol excretion in the tanks of fish that underwent 1) no manipulation, 2) skin swabbing, 3) fin clipping. I believe that the experimental design described in the article is not suitable to provide an answer to the specific research question considered here. Although measuring the cortisol released by a non-manipulated group provided a useful reference value, I believe that there are multiple factors to consider for a more rigorous comparison. 1) Fish handing – it is known that fish netting and air contact are major stress factors that induce an increase of cortisol release in the surrounding water (Ellis, James, Scott 2004). Measuring cortisol concentrations in tanks of non-manipulated fish will very likely indicate lower values, independently from skin swabbing or fin clipping. Hence, a more appropriate control would be represented by fish that underwent the same handling (i.e. netting and transfer to a new tank) without DNA sampling procedure. 2) The pre-procedure treatment of fish that underwent skin swabbing or fin clipping were very different (i.e. 45 minutes exposure to lidocaine in a confined space versus rapid treatment with MS-222). From the results presented here, it is not possible to evaluate whether the 45 minutes pre-treatment with lidocaine did or did not affect cortisol release. In conclusion, this suggests that an experiment aimed at evaluating the stress response to different DNA sampling procedures should include specific negative controls for each treatment type and fish handling procedure.*

**Author Response:**

 The author is right to note that handling fish, and removing them from their tank could affect cortisol release. We have already investigated the constituent steps that make up the swabbing and fin clipping procedures, and these results have been published in two separate papers (Tilley et al., 2020; Tilley et al., 2023). Since the focus of this paper is the step-by-step protocol that to aid uptake of the swabbing technique, we have chosen not to include all of this data here. We have made this point clearer, by modifying the legend to figure 4 (showing the cortisol results) to read “Please refer to Tilley et al., 2020 for further control treatment groups and data points”.

***Reviewer Comment:***

 15. *In the discussion, the authors reported that “changes in gene expression and behaviour caused by skin swabbing tended to appear between two and seven days after manipulation, perhaps due to the activation of an immune response, or changes in ionic or osmotic regulation (Dash et al., 2018)’. In my opinion, this possibility is concerning and should be explored in further studies, as it may hamper any 3Rs benefit of skin swabs. For example, are swabbed fish in the laboratory more susceptible to infection and disease in the longer term compared to fin clipped fish?*

**Author Response:**

 Thank you for this suggestion. We don’t know why we see such as delayed impact of swabbing upon gene expression and behaviour, but we are currently investigating this. We hope to be able to report the effect of swabbing upon the mucus layer in more detail in a future publication.

***Reviewer Comment:***

 16. *The reference de Lombaert et al. (2017) is not present in the reference list.*

**Author Response:**

 Thank you for spotting this error. We have now added de Lombaert et al., 2017 to the reference list.
